# Using service guidance to shape the delivery of cancer services: experience in the UK

**DOI:** 10.1038/sj.bjc.6601079

**Published:** 2003-08-15

**Authors:** R A Haward

**Affiliations:** 1 Leeds University, Leeds, UK

**Keywords:** health care system, oncology care

The development and use of service guidance was one of a number of important United Kingdom (UK) policy initiatives for cancer services introduced over the last 15 years. These initiatives began with the national programme to introduce breast screening in the late 1980s. This was a carefully managed process supported by additional resources. There were new guidelines, specific arrangements for monitoring and evaluation, and ongoing quality management. This well-organised approach to screen-detected disease contrasted sharply with the absence of an equivalent approach to symptomatic breast cancer patients. There was evidence of considerable variability in symptomatic breast services (Chouillet *et al*, 1994; Sainsbury *et al*, 1995; Richards *et al*, 1996) and pressure to address this issue from professional communities (British Breast Group, 1994) and patient groups. At the same time, deeper anxieties were emerging about the performance of UK cancer services. International comparisons suggested that the UK did less well than many comparable countries (Sant *et al*, 1995, 1998). Public, professional and media concerns focused on the fact that when potential cancer patients were referred to hospital, this was not necessarily to a specialist in their particular cancer. Were patients' chances of a good outcome overdependent on their initial hospital referral?

The challenge to look afresh at cancer care was taken up by the Chief Medical Officers in England and Wales, and by their counterparts in Scotland and Northern Ireland. This led to the publication of a policy document generally known as the *Calman-Hine* report (Department of Health, 1995) in 1995, with equivalents in Scotland and Northern Ireland. The evidence supporting this policy was later published in summary form (Selby *et al*, 1996). This was the first ever national policy for the delivery of cancer services in the UK. It offered a clear framework for the delivery of good cancer care, which tried to put the patient at the heart of policy. The issue of specialisation was tackled directly through recommendations for the development of explicit multiprofessional multidisciplinary clinical management arrangements. These were to be available to all patients. *Calman-Hine* had a sustained impact and has been a crucial policy initiative. The positive reactions from many clinicians, the public and NHS organisations to the recommendations implied widespread acceptance of the need to deal with the weaknesses in UK cancer care. It was this broadly based support for change, rather than any particularly effective national strategy for implementation, that enabled action to follow. The one exception was the crucial early decision to commission disease-specific service guidance. This was to complement the overall policy framework by providing detailed recommendations for each of the main types of cancer. The first cancer guidance (all are entitled ‘Improving Outcomes in … Cancer’) covered breast cancer (Department of Health, 1996). The exercise broke new ground, as there was no previous ‘working model’ of national service guidance in the UK. The guidance development process was rigorous and utilised systematic evidence reviews together with the careful involvement of expert opinion (Haward, 1998; Haward and Sheldon, 1999; Haward and Eastwood, 2001). These were complex reports to produce and the supporting evidence reviews are substantial pieces of work. Just as these documents were a challenge to their developers, they posed new questions for managers. The NHS had not previously been faced with implementing such explicit and comprehensive recommendations for the clinical organisation and delivery of services for specific diseases.

The first guidance, for breast cancer, set out the nature and roles expected of multidisciplinary breast cancer care teams. Core team members were specified and each was to be a specialist in breast cancer within their discipline. Each team had to have an adequate caseload (>100 new cases per year) to maintain and develop its expertise and justify the investment in bringing the team together. The simple aim was that all potential breast cancer patients should be assessed, diagnosed and managed by such a team. Similar guidance followed for colorectal and lung cancer. The first three guidance documents recommended that services should be based on local cancer units (Haward, 1995) rather than being concentrated at specialised cancer centres (except for radiotherapy, thoracic and liver surgery), thus laying the groundwork for a coherent approach to service delivery for common cancers. The process of establishing new teams for these common cancers addressed another key recommendation in *Calman-Hine* to expand access to nonsurgical oncologists (clinical and medical oncology) at cancer unit level, with more chemotherapy for these diseases being delivered locally (Haward and Amir, 2000). In England, modest additional funds were allocated to support implementation of each guidance document in turn, promoting dialogue between health organisations and clinicians about priorities (NHS Executive, 1997). This investment signalled political commitment to implementing the service guidance.

Subsequent guidance documents were far more complex as they each dealt with services for groups of cancers rather than single diseases (e.g. gynaecological malignancies). They covered issues common to the group of cancers, such as access and diagnosis, as well as individual site-specific matters (such as staging or treatment). Particular attention was given to integrating care between different parts of the service. The longstanding problems of optimal service configuration were addressed with greater clarity than previous national policies, delineating the respective clinical roles of local units and specialised centres for the diseases in question. They recommended centralisation of specific clinical services such as radical surgical procedures and defined the necessary scale of activity for specialist teams to work effectively and efficiently.

Thus, the guidance addressed a key concern about UK cancer care, the fact that complex clinical work was often very thinly spread, with many patients being treated by individual clinicians or in hospitals managing few cases of any particular type over a 12 month period. While the published evidence that this practice was undesirable is of variable quality, and the issue undoubtedly arouses controversy, results from recent systematic reviews suggest that greater specialisation and higher caseloads lead to better outcomes, particularly for high morbidity or mortality interventions (Hillner *et al*, 2000; Institute of Medicine, 2001; Teisberg *et al*, 2001; Halm *et al*, 2002).

Although it was widely acknowledged that services for intermediate and rare types of cancer needed to be delivered more consistently, with patterns of service delivery reflecting caseload and the complexities of diagnosis and treatment, there was little prior consensus on the way forward. The guidance provided an invaluable ‘route map’ for important reconfigurations in service delivery. It has shifted the NHS debate (or the absence of debate) from what to do, towards active discussion about how best to implement the required changes. Major shifts in patterns of service delivery are inevitably complicated to bring about, and can face professional and institutional resistance to move away from the status quo. Progress in the UK has been hampered by genuine manpower and logistic difficulties in bringing together the requisite people and facilities to operate specialist services on a larger scale. Most key disciplines remain in short supply. It was appreciated at the outset that time would be needed for cancer networks to build up the necessary clinical resources, including expertise across the full range of clinical disciplines, supported by the necessary facilities. To help implementation, the guidance included an economic analysis of the cost implications of these complex service configuration issues, which demonstrated that costs were very variable and dependent on the prior state of local services in the particular field (Ward *et al*, 2000). This emphasised the need for implementation to be dealt with at local level, with priorities based on the actual situation in each place. Resources to support the changes have proved hard to pull together despite the identification nationally of ‘new money’ for investment in cancer services in the National Cancer Plan for England (Department of Health, 2000). This problem has been compounded by the number of health organisations with control over resources in the areas covered by many cancer networks.

Although the *Calman-Hine* report encouraged the concept of networks linking primary care, community services, local hospitals (cancer units) and specialist cancer centres, it offered no practical advice on organisational issues. As a result, cancer networks developed slowly, and were frequently seen by managers as purely clinical in scope. Indeed, it took five more years before concerns about the uneven implementation of cancer policy found coherent expression. The key initiatives in England (with similar but not identical initiatives in the rest of the UK) included the appointment of a National Cancer Director, the publication of a National Cancer Plan and the development of Cancer Service Standards (Department of Health, 2000; NHS Executive, 2000). These standards formed the basis for peer review of cancer services across England, a process that is to be repeated at intervals. Many individual standards were taken directly from the ‘Improving Outcomes’ guidance.

The amount of organisational change affecting the NHS has made it harder for statutory bodies to work together effectively to achieve long-term changes to clinical services. Cancer networks (mandatory rather than statutory organisations) have therefore grown in importance backed by explicit national support for their roles in leading the development of cancer services. This has led them becoming better-resourced organisations despite ambiguities about their organisational status. Successive guidance documents have laid increasing stress on the responsibilities of cancer networks to oversee complex service changes. Their success is crucial if long-term change is to be achieved, yet the control and allocation of resources still requires them to work with and through multiple statutory health organisations in their areas.

A further government initiative to expand the scope of cancer networks was the establishment of an English National Cancer Research Network (NCRN) in 2001. This was preceded by an equivalent exercise in Wales and subsequently mirrored by similar initiatives in Scotland and Northern Ireland. The NCRN has significant resources to promote and support higher rates of entry of cancer patients into clinical trials and other well-designed studies. Cancer networks have received additional investment to enable them to form an associated research network for this purpose. While the primary goal was to expand clinical cancer research, it was also intended to improve cancer care through the more widespread adoption of modern protocols of care. The formation of research networks stimulated clinical collaboration across the hospitals within each cancer network.

The adoption and use of documented clinical policies such as protocols and clinical guidelines can be an important means of achieving consistent standards of care. In Scotland, the long-running series of SIGN clinical guidelines includes guidelines for a number of types of cancer (http://www.sign.ac.uk; Scottish Cancer Therapy Network, 1997). There has been no equivalent initiative in England and Wales until the establishment of the National Institute for Clinical Excellence with a specific role in the production of clinical guidelines. This body now has responsibility for the ‘Improving Outcomes’ series of service guidance, with new titles and updates in preparation. However, there are as yet, no published national clinical guidelines on cancer from NICE although these are planned for the management of genetic risk in familial breast cancer and for lung cancer. NICE have established a new National Collaborating Centre for Cancer, which will begin a long-term programme of clinical guideline development in cancer from April 2003. Around the time of the *Calman-Hine* report, limited encouragement was given to professional bodies with interests in cancer to publish clinical guidelines and a number have done so successfully, although there was no consistency in the methodologies used (British Association of Surgical Oncology, 1995; Royal College of Surgeons of England & Association of Coloproctology of Great Britain and Ireland, 1996).

At local level, there has been interest in using clinical guidelines to bring clinical networks together and provide a rational basis for audit. While evidence-based service guidance has provided the route map for improving the organisation of cancer care, it has not been complemented by an equally convincing approach to the development and use of clinical guidelines, outside Scotland. It seems self-evident that there must be opportunities for greater cooperation and collaboration in the preparation and use of clinical guidelines within the UK and between the UK and other countries with similar interests. Systematic evidence reviews and reputable processes of guideline development are expensive, and the expertise to do them well is scarce. It is arguable that such investments could be utilised more effectively if guideline developments covered larger clinical communities.

So what conclusions can be drawn about the roles of evidence-based service guidance and clinical guidelines in policies for improving cancer care in the UK? The cancer policy initiatives in the mid-1990s have been of immense importance. They led to a continuous process of cancer services development based on a specialist multidisciplinary model for the delivery of services. The ‘Improving Outcomes Guidance’ played a crucial role, building on the success of the *Calman-Hine* policy, but extending it by specifying how services for particular cancer sites should be delivered. Reconfiguration of services for the intermediate or rarer cancers would not have been approached consistently – if at all, had evidence-based guidance not been developed, and remains dependant on the framework of national service guidance. The potential of authoritative clinical guidelines to promote consistent clinical policies and practice has not been sufficiently exploited outside Scotland.

Implementation of cancer policy was handled differently in the various UK countries, and was inconsistent within England. Policy implementation as well as policy development must be addressed coherently if any strategy is to succeed in changing services in fact rather than in theory. Access to resources to support desired changes has been a critical issue and tensions remain between the governments' disease-specific developmental priorities – aiming for long-term improvements in the way complex clinical services are delivered (and ultimately in the outcomes they achieve) and other competing priorities set for the NHS. The devolution of control over resources to large numbers of statutory organisations makes it harder to coordinate developments requiring investment in secondary- and tertiary-level hospital services.
